# High-throughput sequencing and characterization of potentially pathogenic fungi from the vaginal mycobiome of giant panda (*Ailuropoda melanoleuca*) in estrus and non-estrus

**DOI:** 10.3389/fmicb.2024.1265829

**Published:** 2024-01-25

**Authors:** Xiaoping Ma, Zhen Liu, Chanjuan Yue, Siwen Wang, Xinni Li, Chengdong Wang, Shanshan Ling, Ya Wang, Songrui Liu, Yu Gu

**Affiliations:** ^1^Key Laboratory of Animal Disease and Human Health of Sichuan Province, College of Veterinary Medicine, Sichuan Agricultural University, Chengdu, China; ^2^Chengdu Research Base of Giant Panda Breeding, Sichuan Key Laboratory of Conservation Biology for Endangered Wildlife, Sichuan Academy of Giant Panda, Chengdu, China; ^3^China Conservation and Research Center for the Giant Panda, Chengdu, China; ^4^College of Life Sciences, Sichuan Agricultural University, Chengdu, China

**Keywords:** giant panda, fungi, high-throughput sequencing, isolation and identification, pathogenicity

## Abstract

**Introduction:**

The giant panda (*Ailuropoda melanoleuca*) reproduction is of worldwide attention, and the vaginal microbiome is one of the most important factors affecting the reproductive rate of giant pandas. The aim of this study is to investigate the diversity of vaginal mycobiota structure, and potential pathogenic fungi in female giant pandas during estrus and non-estrus.

**Methods:**

This study combined with high-throughput sequencing and laboratory testing to compare the diversity of the vaginal mycobiota in giant pandas during estrus and non-estrus, and to investigate the presence of potentially pathogenic fungi. Potentially pathogenic fungi were studied in mice to explore their pathogenicity.

**Results and discussion:**

The results revealed that during estrus, the vaginal secretions of giant pandas play a crucial role in fungal colonization. Moreover, the diversity of the vaginal mycobiota is reduced and specificity is enhanced. The abundance of *Trichosporon* and *Cutaneotrichosporon* in the vaginal mycobiota of giant pandas during estrus was significantly higher than that during non-estrus periods. *Apiotrichum* and *Cutaneotrichosporon* were considered the most important genera, and they primarily originate from the environment owing to marking behavior exhibited during the estrous period of giant pandas. *Trichosporon* is considered a resident mycobiota of the vagina and is an important pathogen that causes infection when immune system is suppressed. Potentially pathogenic fungi were further isolated and identified from the vaginal secretions of giant pandas during estrus, and seven strains of *Apiotrichum* (*A. brassicae*), one strain of *Cutaneotrichosporon* (*C. moniliiforme*), and nine strains of *Trichosporon* (two strains of *T. asteroides*, one strain of *T. inkin*, one strain of *T. insectorum*, and five strains of *T. japonicum*) were identified. Pathogenicity results showed that *T. asteroides* was the most pathogenic strain, as it is associated with extensive connective tissue replacement and inflammatory cell infiltration in both liver and kidney tissues. The results of this study improve our understanding of the diversity of the vaginal fungi present in giant pandas and will significantly contribute to improving the reproductive health of giant pandas in the future.

## 1 Introduction

The giant panda (*Ailuropoda melanoleuca*) is a flagship species of global biodiversity conservation, and has been described as a “living fossil” and a “national treasure of China.” Despite the comprehensive efforts of biologists to increase its population in recent years, the Giant Panda was graded as vulnerable by the International Union for Conservation of Nature in 2016, and the reproduction of giant pandas has drawn worldwide attention (Ma et al., 2018). The vaginal microbiota is thought to be an important factor affecting the reproductive rate of giant pandas (Yang et al., 2017).

The vaginal microbiome is an important microbiota community in the mucous membrane of the reproductive tract that affects the physiology, reproduction, and health of the host and acts as a barrier against pathogen invasion (Smith and Ravel, 2017). An unbalanced mycobiota and vaginal infections have been shown to be significantly associated with infertility (Li et al., 2019). Common infections of the reproductive system, such as fungal vaginitis and cervicitis, can reduce pregnancy success in animals and even increase the risk of abortion. Fungi have been isolated from the reproductive tracts of animals since Smith first isolated them from cattle uteri in 1920 (Smith, 1920). Fungal abortion caused by *Aspergillus* has also been reported in dairy cows (Henker et al., 2022). Fungal infections cause endometritis and cervicitis in horses (Orellana-Guerrero et al., 2019). Numerous reports have documented skin diseases and invasive infections in giant pandas caused by fungi, impacting their growth and development and, in severe cases, leading to death (Chen, 2005; Ma et al., 2019). Chen et al. (2020) first reported mycotic vaginitis caused by a *Candida albicans* infection in postpartum giant pandas.

Fungi are widely found in nature, water, air, and soil, and participate in myriad ecological functions (Spiteller, 2015). According to the behavioral ecology of giant pandas, the vulva swells during estrus, turns outward, and the marking behavior of perianal gland markers increases, which can increase contact between the vulva and the external environment, thereby increasing the chances of fungi entering the vagina (Ma et al., 2022). The traditional classification of fungi is mainly based on their morphology, growth, and physiological and biochemical characteristics. However, many fungi grow under harsh conditions and cannot be cultured under these conditions. High-throughput and metagenomic sequencing have become important methods for detecting microbiota diversity and can more accurately interpret complete fungal genomes (Grewal et al., 2021). Zhang et al. (2020) conducted metagenomic sequencing of the vaginal secretions of giant pandas during estrus and among the top 35 genera found seven fungal genera, all of which were basidiomycetes (Zhang et al., 2020). Using high-throughput sequencing, Chen et al. (2018) analyzed the vaginal mycobiota of giant pandas of various ages and under different living environments and found that the dominant genera were *Guehomyces* (37.92%), *Cladosporium* (9.072%), *Trichosporon* (6.2%), and *Mucor* (4.97%) (Chen et al., 2018). Ma et al. (2017) assessed healthy giant panda vaginal samples during estrus, primarily isolating *Aspergillus* (10.53%), *Trichosporon* (9.21%), and *Penicillium* (6.58%), and identified *Trichosporon japonica* and *Trichosporon asteroides* from giant pandas for the first time at the species level. To date, there have been relatively few studies on the vaginal fungal communities of giant pandas, and most of the available reports have focused on the effects of the environment and age. However, comparisons of the differences in the structures of vaginal fungal communities of giant pandas during estrus and non-estrus periods have not been reported.

This study aimed to explore the differences in vaginal mycobiota structure and potential pathogenic fungal composition between the estrus and non-estrus stages of giant pandas using high-throughput ITS gene sequencing. Potentially pathogenic fungi were isolated and identified from the vaginal secretions of giant pandas during estrus using laboratory tests. In addition, mice were used as infection models to explore the pathogenicity of fungi. Exploring the mycobiome of giant panda vaginal fungi can help provide a better understanding of the diversity of giant panda vaginal fungi. Further, identifying potentially pathogenic fungi is of great significance for improving the reproductive health of giant pandas in the future.

## 2 Materials and methods

### 2.1 Sample collection

Samples were collected from September 2019 to April 2020 at the Chengdu Research Base of Giant Panda Breeding (Chengdu, Sichuan) and the Wolong China Giant Panda Garden (Aba Tibetan and Qiang Autonomous Prefecture, Sichuan). Vaginal swabs were collected from 15 adult female giant pandas at the Chengdu Research Base of Giant Panda Breeding, including nine vaginal samples (EV1-EV9) of giant pandas in estrus (EV) and six vaginal samples of non-estrous giant pandas (NEV) named NEV1-NEV6. All 15 samples were subjected to high-throughput sequencing, and the nine samples from pandas in estrus were retained for further laboratory testing. Vaginal swabs were collected from nine adult female pandas (WL1-WL9) during estrus at the Wolong Panda Breeding Center for laboratory testing. Anesthesia, sampling, and other animal procedures were approved by the Chengdu Research Base of Giant Panda Breeding (No. 2019006) and the Wolong China Giant Panda Garden (No. 2019403033) Institutional Animal Care and Use Committee (IACUC). Samples were collected from female giant panda during both estrus and non-estrus periods, determined by the month of estrus and the detection of estrogen in their body (Cai et al., 2017).

All sampling procedures were aseptic, performed with disposable sterilized caps, masks, gloves, and protective clothing worn. During sampling, the vaginas of giant pandas were opened moderately with a sterilized vaginal dilator, and a 20 cm-long sterile cotton swab was inserted into the vagina and rotated repeatedly along the vaginal wall to collect vaginal secretions as samples. Samples for high-throughput sequencing were delivered to the laboratory and kept in a thermostat at 4°C for approximately 5 ~ 10 min and then immediately stored in a freezer at −80°C. All samples were stored on dry ice after collection for delivery. The vaginal secretions of giant pandas, for isolation and culture, were collected and placed in a 4°C car incubator and sent to the laboratory within 2 h for isolation and culture. A summary of the giant pandas is presented in Supplementary Table S1.

### 2.2 DNA extraction, PCR amplification, and its sequencing

Fifteen vaginal secretion samples from giant pandas were sent to BGI Co., Ltd. (Shenzhen, China) for DNA extraction and ITS gene sequencing analysis. Fungal DNA was extracted according to the manufacturer’s instructions (BGI Co., Ltd., Shenzhen, China). The fungus-specific primers used for amplifying the ITS region were ITS1 (5’-TCCGTAGGTGAACCTGCGG-3′) and ITS4 (5’-TCCTCCGCTTATTGATATGC-3′) (White et al., 1990). The total amplification volume of PCR was 25 μL, consisting of 1 μL of forward and reverse primers, 12.5 μL of PCR master mix, 30 ng of template DNA, and water added to make 25 μL. Reaction procedure: initial denaturation at 94°C for 10 s, followed by denaturation at 94°C for 30 s, annealing at 55°C for 30 s, extension at 72°C for 1 min, 30 cycles, and final extension at 72°C for 5 min. The PCR amplification product was purified using Encourt AMPure XP magnetic beads for library construction. The fragment range and concentration of the library were determined using the Agilent 2,100 bioanalyzer. Qualified libraries were sequenced using the HiSeq2500 platform.

### 2.3 Fungal isolation and identification

The swab tube was pretreated with 2 mL of sterile distilled water. Sample swabs were inoculated on Sabouraud dextrose agar (SDA) medium using disposable inoculation rings and cultured at 25°C for 5–8 days to observe colony growth. Fungal colonies with different morphologies and colors were isolated until a single colony was obtained by purification. Amplification of the ITS region, D1/D2 domain, and IGS1 region was performed as described with the primer pairs ITS1/ITS4 (ITS1:5′-TCCGTAGGTGAACCTGCGG-3′; ITS4:5′-TCCTCCGCTTATTGATATGC-3′), F63/R635 (F63:5′-GCATATCAATAAGCGGAGCAAAAG-3′; R635:5′-GGTCCGTGTTTCAAGACG-3′), and 26SF/5SR (26SF,5′-ATCCTTTGCAGACGACTTGA-3′, 5SR:5′-AGCTTGACTTCGCAGATCGG-3′), respectively (Diaz and Fell, 2004). The total amplification volume of PCR was 50 μL, consisting of 1 μL of forward and reverse primers, 25 μL of PCR master mix, 3 μL of fungal genomic DNA, and water added to make 50 μL. Reaction procedure: initial denaturation at 94°C for 10 min, followed by denaturation at 94°C for 30 s, annealing at 55°C for 30 s, extension at 72°C for 10 s, 30 cycles, and final extension at 72°C for 4 min. The PCR products were sent to Youkang Biotech Co., Ltd. (Chengdu, China) for sequencing and NCBI BLAST was performed on the sequencing data. Phylosuite software was used to concatenate the ITS, IGS1, and D1/D2 genes, and the IQ-Tree method was used to construct a phylogenetic tree based on the maximum likelihood method (Xiang et al., 2023); *Cladosporium cladosporioides* CBS 112388 served as an outgroup.

### 2.4 Growth of fungi and conidia preparation

Purified fungi were isolated, washed with sterile phosphate buffered saline (PBS), and transferred to sterile centrifuge tubes. The conidia were diluted with PBS to 1 × 10^6^ and 1 × 10^7^ colony forming units (CFU)/mL using a hemocytometer.

### 2.5 Morphological studies

Microscopic observation of the isolates was performed after slide culture on SDA. A 0.5 mL of melted SDA was injected into a closed glass Petri dish composed of a slide glass, a cover glass, and a copper ring with a hole in the wall (Feng et al., 2014). A 5 μL sample of the conidial suspension (1 × 10^6^ CFU/mL) was inoculated through the hole of the copper ring. All isolates were incubated at 25°C and observed after 24, 48, 72, and 96 h. The cover glass of the closed glass Petri dish was removed, stained with 5 μL of lactophenol cotton blue (Hopebio, Qingdao, China) and observed under a microscope (BX51, Olympus).

### 2.6 Animals and experimental infection

Specific pathogen-free 6-week-old female mice of the KM strain (Dashuo Experimental Animals Co., Ltd. Chengdu, China) were inoculated with fungus to construct an infection model. The mice were euthanized using the neck-breaking method, and the lesions were observed anatomically. All animal experiments were approved by the Institutional Animal Care and Use Committee of Sichuan Agricultural University (permit number DYY-S20183033).

The experiment was divided into seven groups, including six experimental groups and one control group. Each experimental group consisted of immunosuppressed and non-immunosuppressed groups. Each group comprised three mice. Mice in the immunosuppressed and non-immunosuppressed groups were intraperitoneally injected with 0.1 mL of a 1 × 10^7^ CFU/mL conidial suspension, while mice in the control group were intraperitoneally injected with 0.1 mL of PBS, with three mice in each group. Feeding and clinical symptoms of the mice were observed daily. Mice in the immunosuppressed group were intraperitoneally injected with 50 mg/kg cyclophosphamide (Jiangsu Hengrui Pharmaceutical Co., Ltd., Lianyungang, China) and 15 mg penicillin sodium (Jiangsu Hengrui Pharmaceutical Co., Ltd., Lianyungang, China) once a day for immunosuppression, 3 days before infection with the conidial suspension.

### 2.7 Histopathological analysis

Liver and kidney tissues of mice inoculated with the conidial suspension for 7 days were collected and fixed at 4% (v/v) for routine treatment with buffered formalin and paraffin embedding. The samples were stained with hematoxylin and eosin (HE) for histopathological evaluation and periodic acid-Schiff (PAS) staining to assess fungal invasion in the tissue structures.

### 2.8 Statistical analyses

The raw data was filtered using the following methods to eliminate adapter contamination and low-quality readings. Using the software FLASH (Fast Length Adjustment of Short reads, v1.2.11), the paired reads obtained by double-terminal sequencing were assembled into a sequence by using the overlapping relationship to obtain the tags of the highly variable region (Magoč and Salzberg, 2011). Divisive Amplicon Sequence Variants (ASVs) were obtained using the DADA2 (Divisive Amplicon Denoising Algorithm) function in the QIIME2 software, and the ASVs were 100% similar. The feature list and sequences were then obtained. Alpha and beta diversities were analyzed based on the feature sequences and feature lists. The QIIME2 plug-in feature classifier was used to compare species annotation sequences, and the Ribosomal Database Project (RDP) and Unite were used for comparison (Caporaso et al., 2010).

Venn plots, stacked bars, and sparse curves for each group were displayed using R software (v3.1.1). Alpha diversity indices between groups were compared by performing a Wilcox test using R software (v3.2.1). UPGMA cluster analysis of the weighted Unifrac distance matrix was performed using phytools and R software (v3.5.1), to visualize the species compositions of the samples and how they differ. Differences in the top 10 fungi, in abundance, between the EV and NEV groups were tested for statistical significance by R software (v3.4.1). The Wilcox test was used to count the relative abundance of the species in each group, and the Kruskal-Wallis test was used to count the average relative abundance of the species in each group. LEfSe software was used to plot the LEfSe cluster diagram and LEfSe linear discriminant analysis (LDA) diagram. In this study, *p* < 0.05 indicates a significant difference (*p < 0.05).

## 3 Results

### 3.1 Sequencing data

**Figure 1:**
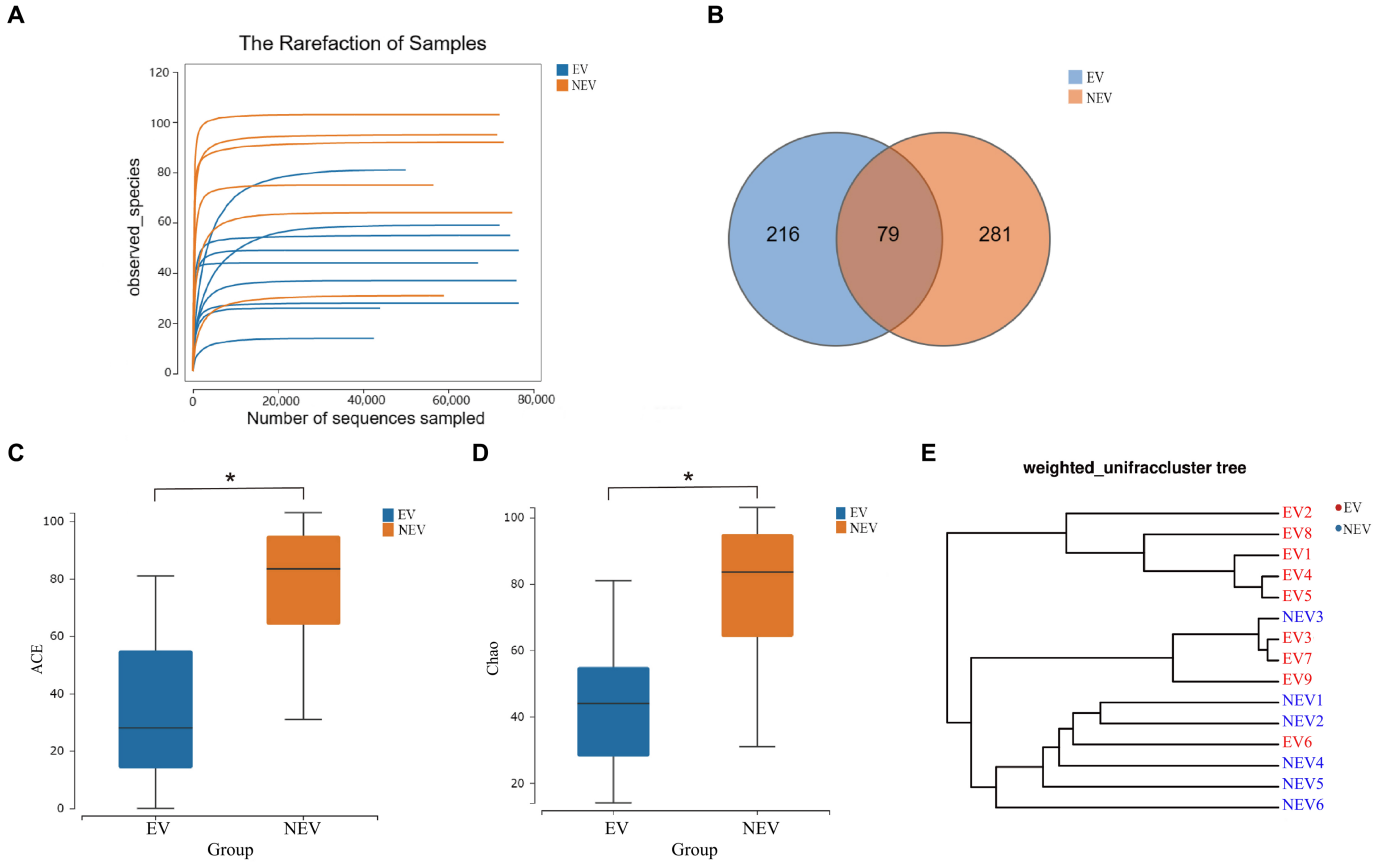
**(A)** Rarefaction curve. The observed species (Y-axis) of each sample at different numbers of sequences sampled (X-axis). **(B)** Venn diagram. Distribution of amplicon sequence variants (ASVs) in the estrus vaginal (EV) and non-estrus vaginal (NEV) groups. **(C)** Comparison of ACE diversity indices. **(D)** Comparison of Chao1 diversity indices. **(E)** Results from sample clustering. Most samples in the same group have similar branches (*p < 0.05).

After quality control, 989,978 sequences were generated from the EV and NEV groups. Sparse curves were used to calculate the sequencing quality. All curves were flat, and the number of ASVs was near saturation (Figure 1A), indicating that the sequencing volume of all samples was sufficient to reflect most of the fungal diversity information. In total, 576 ASVs were identified, of which 79 were shared between the two groups, with 216 and 281 unique ASVs in the EV and NEV groups, respectively (Figure 1B).

### 3.2 ASV abundance analysis

The ACE and Chao1 indices were positively correlated with the number of species in the community and were used to estimate alpha diversity (Figures 1C,D). The ACE and Chao1 indices of the NEV group were significantly higher than those of the EV group (*p* < 0.05), indicating that species richness of the NEV group was significantly higher than that of the EV group. UPGMA cluster number results were used to demonstrate beta diversity. UPGMA cluster number results showed that most samples in the same group had similar branches (Figure 1E). The fungal communities in the EV and NEV groups showed an obvious aggregation pattern, indicating that the vaginal mycobiota in estrus is significantly different from that in non-estrus.

### 3.3 Community-composition analysis

**Figure 2:**
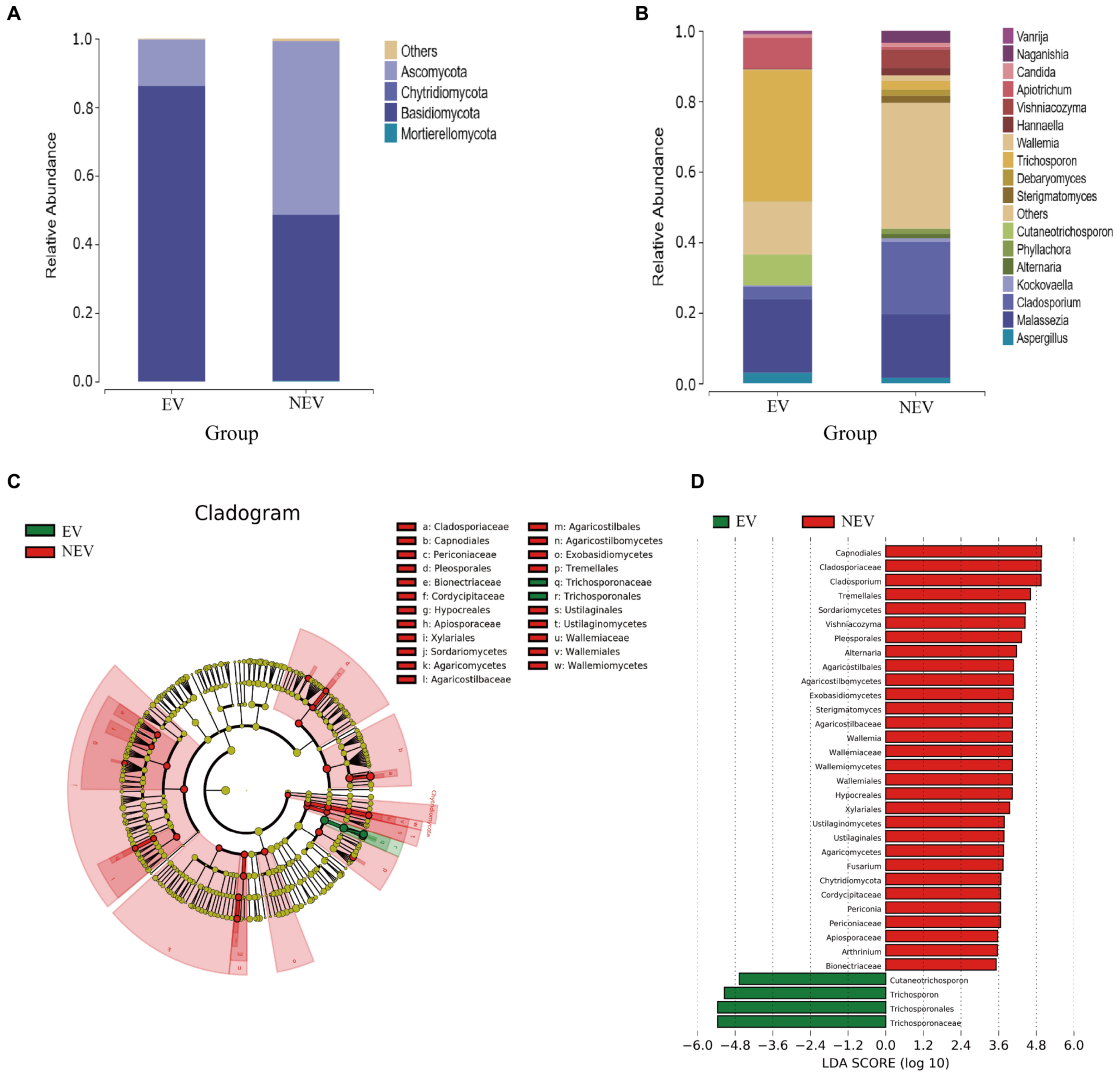
Bar graphs showing the composition of fungal species and abundances detected in the EV and NEV groups at the level of phylum **(A)** and genus **(B)**. **(C)** LEfSe clustering tree. Green nodes and red nodes represent the important mycobiome in the estrus vaginal (EV) and non-estrus vaginal (NEV) groups, respectively. From the inner circle and moving outwards, the circles represent taxa at the level of phylum, class, order, family and genus. **(D)** Linear discriminant analysis (LDA) diagram, showing only significantly different species with an absolute LDA score value greater than 2. The EV and NEV groups have significant influence on the fungal groups. The LDA score represents the size of the impact of significantly different species between different groups.

The fungi composition of the 15 samples was analyzed, and the average relative abundance at the gate level was calculated. Four core gates were identified at the phylum level: *Ascomycota*, *Chytridiomycota*, *Basidiomycota* and *Mortierellaomycota*. *Ascomycota* and *Basidiomycota* were the most common dominant groups in the EV and NEV groups, accounting for 96.18 and 98.85% of the EV and NEV groups, respectively, indicating that the composition of giant panda vaginal fungi in the two groups was similar at the phylum level. In addition, *Chytridiomycota* and *Mortierellaomycota* were detected only in the NEV group, suggesting that the colony diversity in the NEV group was higher than that in the EV group (Figure 2A).

At the genus level, the core genera included *Trichosporon* (19.86%), *Malassezia* (19.47%), *Cladosporium* (10.31%), *Apiotrichum* (4.78%), *Cutaneotrichosporon* (4.38%), and a total of 17 genera were identified, accounting for 79.72% of the composition of all samples (Figure 2B). *Malassezia* (38.93%), *Trichosporon* (37.26%), *Apiotrichum* (8.85%), *Cutaneotrichosporon* (8.69%), *Cladosporium* (3.49%), *Aspergillus* (3. 04%) were the predominant taxa in the EV group. *Cladosporium* (20.59%), *Malassezia* (18.05%), *Vishniacozyma* (5.28%), *Naganishia* (3.38%), and *Trichosporon* (2.45%) were the predominant taxa in the NEV group (Figure 2B). The abundances of *Apiotrichum* (0.72%) and *Cutaneotrichosporon* (0.072%) were very low in the NEV group (Figure 2B).

### 3.4 LEfSe analysis

A branch diagram of LEfSe analysis is shown in Figure 2C. Green and red nodes in the branch plot indicate fungi that play key roles in the EV and NEV groups, respectively. The yellow nodes indicate the fungal groups that did not play an important role in the different groups. The linear discriminant analysis (LDA) scores represent the magnitude of the influence of significantly different species between the groups (Figure 2D). At the genus level, *Cladosporium*, *Vishniacozyma*, *Alternaria*, *Wallemia*, *Fusarium*, *Periconia*, *Arthrinium*, and *Strigmatomyces* were significantly different in the NEV group. Only *Trichosporon* and *Cutaneotrichosporon* were significantly different in the EV group. The LDA scores represent the magnitude of the influence of significantly different species between the groups (Figure 2D).

### 3.5 Analysis of species differences

**Figure 3:**
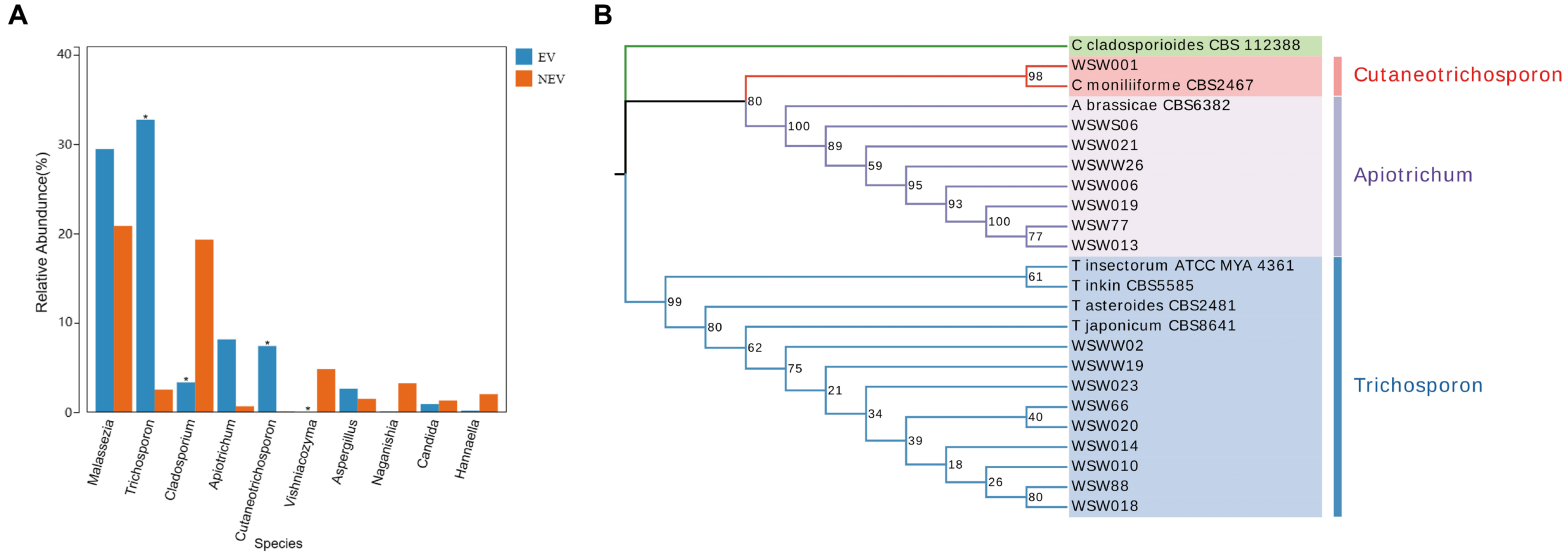
**(A)** Species difference analysis. The top ten most abundant species were selected to show the mean relative abundance of each group and the significance of the difference test (*p < 0.05). **(B)** Phylogenetic tree of *Apiotrichum*, *Cutaneotrichosporon* and *Trichosporon* based on ITS, IGS1, and D1/D2 genes.

The results of the differential species analysis showed that *Trichosporon* and *Cutaneotrichosporon* were significantly higher in the NEV group (*p* < 0.05), whereas *Cladosporium* and *Vishniacozyma* were significantly lower (*p* < 0.05) (Figure 3A). Combining these results with those from the community composition and LEfSe analyses leads to the suggestion that the vaginal colonization by *Trichosporon*, *Cutaneotrichosporon*, *Cladosporium*, and *Vishniacozyma* was seriously affected by giant panda estrus.

### 3.6 Molecular identification

Seventeen strains of potentially pathogenic fungi were isolated from the vaginal samples of eight giant pandas (Table 1). There were seven strains of *Apiotrichum brassicae* (*A. brassicae*), five of *Trichosporon japonicum* (*T. japonicum*), two of *Trichosporon asteroides* (*T. asteroides*), one of *Cutaneotrichosporon moniliiforme* (*C. moniliiforme*), one of *Trichosporon inkin* (*T. inkin*), and one of *Trichosporon insectorum* (*T. insectorum*). A total of 51 DNA sequences of the ITS, IGS1, and D1/D2 regions were obtained by PCR amplification and electrophoretic sequencing and compared with the NCBI BLASTN database. These strains (*A. brassicae*, *T. japonicum*, *T. asteroides*, *C. moniliiforme*, *T. inkin*, *T. insectorum*) were successfully identified and submitted to the GenBank database of NCBI and corresponding serial numbers were obtained (Supplementary Table S2).

**Table 1:** The species isolated from each giant panda individual.

Name	Count	Molecular identification
WL1	1	*A. brassicae*
WL2	1	*A. brassicae*
WL3	1	*A. brassicae*
WL4	1	*T. inkin*
WL5	4	*A. brassicae, T. asteroides, T. japonicum* 2
EV2	1	*C. moniliiforme*
EV4	6	*A. brassicae* 3*, T. japonicum, T. asteroides, T. insectrum*
EV9	2	*T. japonicum* 2

The number after the strain represents the number of isolates.

Because there were no submitted IGS sequences for *T. insectorum* and *T. japonicum* in the NCBI database, only the ITS and D1/D2 regions were concatenated to construct the phylogenetic tree. The phylogenetic tree showed that WSW77, WSW013, WSW019, WSW021, WSW006, WSW026, and WSWS06 are highly homologous to *A. brassicae* CBS6382. The isolated strain, WSW001, showed 98% homology with *C. moniliiforme* CBS2464 (Figure 3B). The isolated strains of *Trichosporon* spp. had similarity with each other and with other *Trichosporon* spp. sequences in the NCBI database, but the overall support rate was low, which may be due to the absence of IGS sequences; therefore, more accurate comparisons could not be made. Our results fill this gap in the literature.

### 3.7 Morphological development process

**Figure 4:**
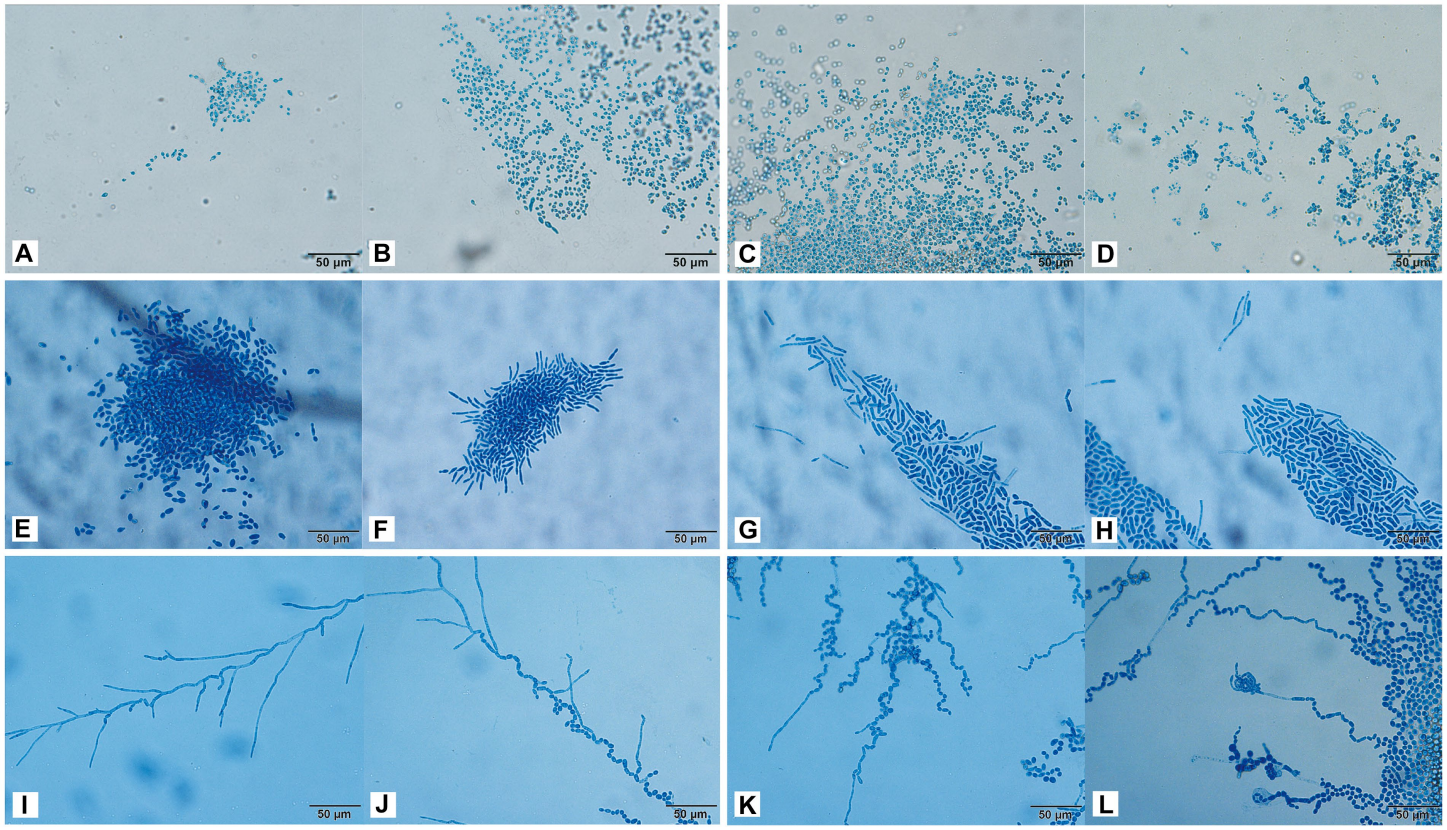
Morphological development process of *C. moniliiforme* **(A–D)**, *A. brassicae* **(E–H)**, *T. asteroides* **(I–L)** from day 1 to day 4. **(A–D)** Micrographs show oval-shaped spores, and no hyphae; **(E)** a large number of oval conidia; **(F–H)** conidia expanded and formed parallel hyphae; **(I)** pseudohyphae radiate; **(J)** Production of conidia and arthrospores; **(K,L)** new conidia sprouting from joint segments. Scale bars, 50 μm.

The conidia of *C. moniliiforme* split spontaneously and developed multilaterally. Moreover, conidia occurred singly or in pairs, sometimes in short chains and clusters, and no hyphae were observed (Figures 4A–D). After 24 h of *A. brassicae* culture, many oval conidia were observed (Figure 4E), and these expanded and formed hyphae by 48 h of culture (Figure 4F). Hyphae were distributed in parallel segments, and they differentiated into spindle-shaped conidia (Figures 4F–H). Furthermore, many slender pseudohyphae were formed after 24 h of *T. asteroides* culture (Figure 4I). The pseudohyphae were radially dispersed with blastospores and arthrospores (Figure 4J). In addition, sporulation was abundant, and the conidia were elliptical. Finally, pseudohyphae nodes sprouted and arthrospores were divided to produce new conidia (Figures 4K,L).

**Figure 5:**
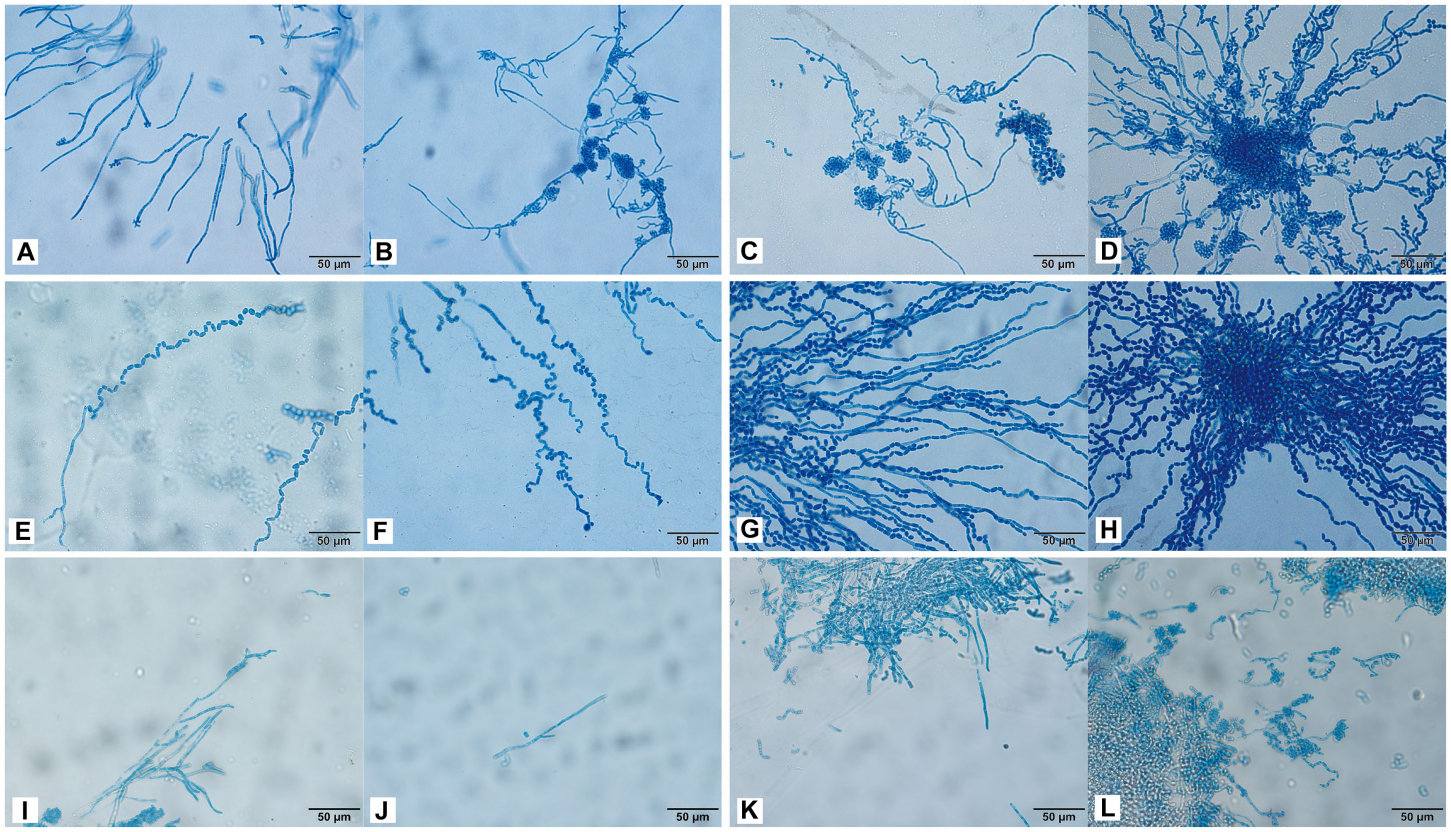
Morphological development process of *T. inkin* **(A–D)**, *T. insectorum* **(E–H)**, and *T. japonicum* **(I–L)** from day 1 to day 4. **(A)** A large number of septate hyphae and some hyphae tips appear antler-like; **(B–D)** a large number of oblong arthrospores, blastospores, and antler hyphae; **(E)** hyphae are differentiated into arthrospores; **(F–H)** the arthrospores form a single oval conidium; **(I)** many pseudohyphae; **(J)** hyphal budding forms near blastospore; **(K,L)** hyphal fragmentation produces a large number of arthrospores. Scale bars, 50 μm.

*Trichosporon inkin* cultured for 24 h produced many septate hyphae (Figure 5A). After 48 h of culture, many oblong arthrospores and blastospores appeared, and most of the blastospores were oval and aggregated into balls (Figure 5B). The hyphal conidia were uniformly stained, and the number of antler hyphae gradually increased with the culture time (Figures 5A–D). The conidia of *T. insectorum* were ovoid, and the hyphae differentiated into arthrospores after cultivation for 24 h (Figure 5E). Pseudohyphae and arthropod conidia were then developed, and the arthrospores formed a single oval conidium (Figures 5F–H). *Trichosporon insectorum* pseudohyphae and articular conidial development occurred throughout the entire detection period, with articular conidia being the dominant morphology (Figures 5E–H). Further, many pseudohyphae were observed after 24 h of *T. japonicum* culture, and budding resulted in the formation of round blastospores (Figures 5I,J). In addition, the hyphae were segmented and associated with the node conidia (Figures 5I–K), and hyphal fragmentation produced many arthrospores after 48 h of culture (Figures 5K,L).

### 3.8 Pathogenicity

We obtained a total of 17 isolates, and here, the isolates of the same species from different individual giant pandas were counted as different isolates. These 17 isolates belonged to six different fungal species throughout the study. Only one isolate of the same fungal species was selected for testing. To explore the pathogenicity of these potentially pathogenic fungi, *C. moniliiforme*, *A. brassicae*, *T. asteroides*, *T. inkin*, *T. insectorum*, and *T. japonicum* were inoculated into mice. Three days after being inoculated with *T. asteroides*, the mice in the immunosuppressed group experienced a significant decrease in activity and appetite, and half of the mice died. Mice in the non-immunosuppressed group had a poor mental state; however, all of them survived. All other inoculated groups of mice exhibited decreased activity, maintained a normal appetite, and did not show any other major changes. One week later, all mice were sacrificed by cervical dislocation and dissected. It was found that the mice inoculated with *T. asteroides* showed massive ascites (Supplementary Figure S1A) and white nodules in the liver (Supplementary Figure S1B). The mice infected with other fungi showed no obvious abnormalities.

To further understand the pathological changes after infection, histological analysis was performed on the kidneys and livers of mice inoculated with *C. moniliiforme*, *A. brassicae*, *T. asteroides*, *T. inkin*, *T. insectorum*, and *T. japonicum*.

**Figure 6:**
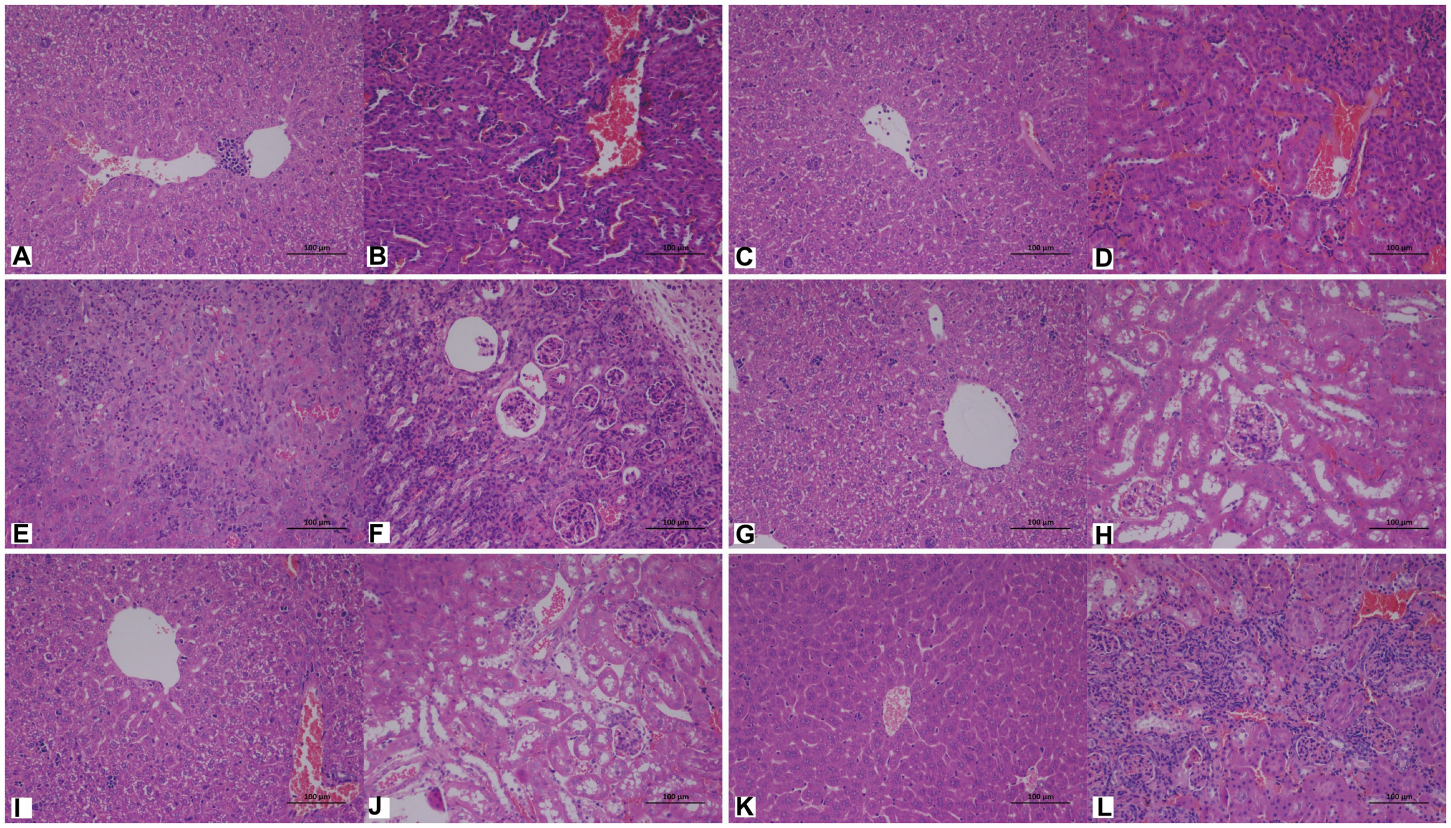
Representative hematoxylin and eosin (HE)-stained sections of liver **(A,C,E,G,I,K)** and kidney **(B,D,F,H,J,L)** tissues of immunosuppressed mice on the 7th day after infection with *C. moniliiforme*, *A. brassicae*, *T. asteroides, T. inkin*, *T. insectorum*, and *T. japonicum*, respectively. The spores and mycelia are indicated with arrows. Scale bars, 100 μm.

**Figure 7:**
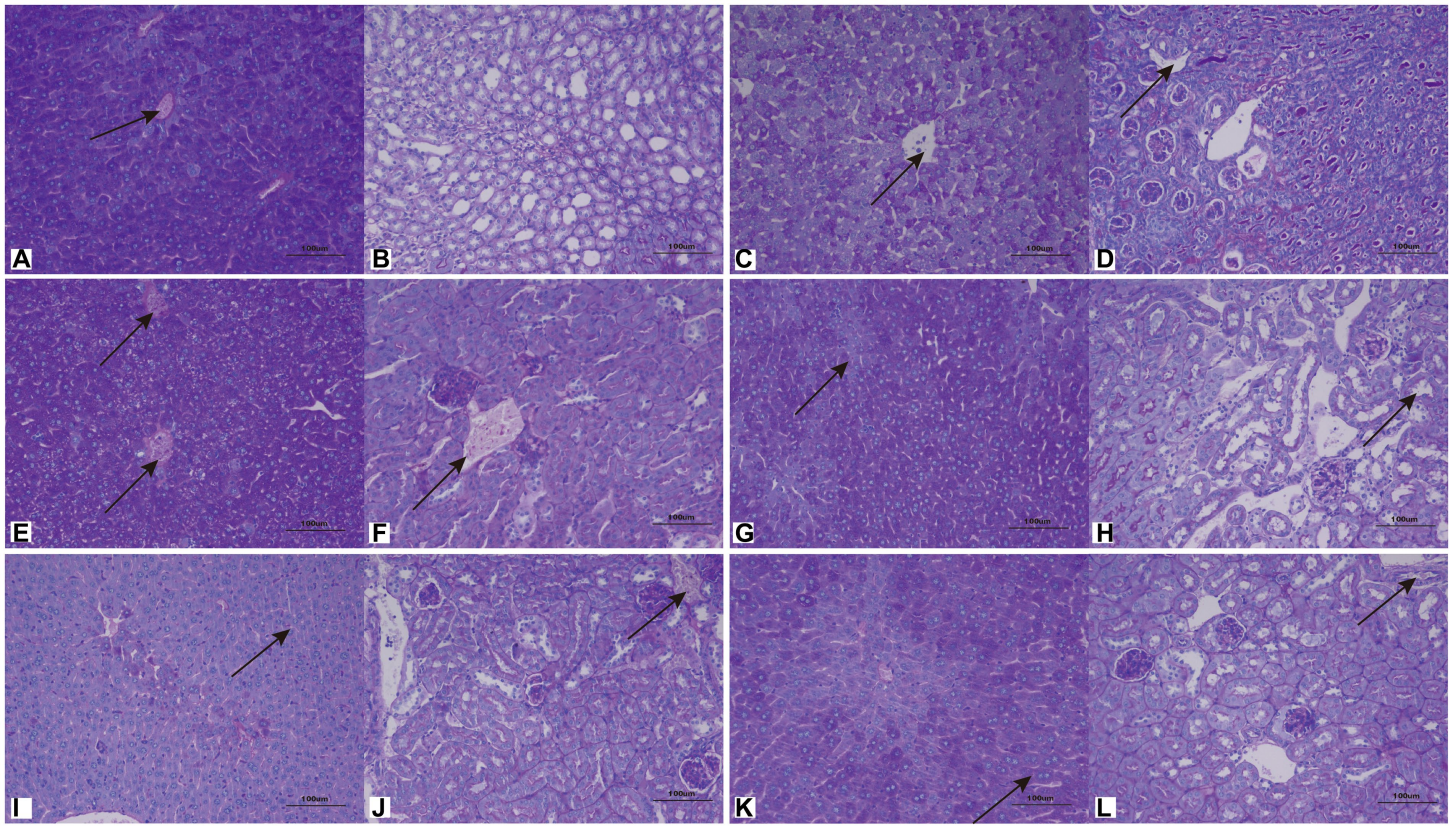
Representative periodic acid–Schiff (PAS)-stained sections of liver **(A,C,E,G,I,K)** and kidney **(B,D,F,H,J,L)** on the 7th day after infection with *C. moniliiforme*, *A. brassicae*, *T. asteroides, T. inkin*, *T. insectorum*, and *T. japonicum*, respectively. The spores and mycelia are indicated with arrows. Scale bars, 100 μm.

*Cutaneotrichosporon moniliiforme* caused slight congestion in the livers of mice, a large amount of liver cell degeneration, loose and light staining of cytoplasm, and a small amount of local lymphocyte infiltration (Figure 6A; Supplementary Figure S2A). A large amount of vascular congestion was observed in renal sections (Figure 6B). The immunosuppressed group (Figures 6A,B) had obvious lesions, whereas the non-immunosuppressed group (Supplementary Figures S2A,B) had insignificant lesions. Mycelia and spores were observed in the liver tissue after PAS staining, but no spores or mycelia were observed in the kidney (Figures 7A,B).

*Apiotrichum brassicae* caused extensive hepatocyte degeneration, increased intercellular space, hepatocyte swelling, loose and light staining of cytoplasm, and a small amount of granulocytic infiltration (Figure 6C). A large amount of vascular congestion, a small amount of renal cell degeneration, and lymphocyte infiltration were observed in the visual field of the kidney section (Figure 6D). The lesions of the immunosuppressed group were more obvious (Figures 6C,D), while the liver and kidney tissues of the non-immunosuppressed group showed no significant pathological changes (Supplementary Figures S2C,D). After PAS staining, spores were found in both liver and kidney tissue (Figures 7C,D).

*Trichosporon asteroides* caused local hepatocytes to be replaced by proliferative connective tissue, accompanied by extensive granulocyte infiltration (Figure 6E), extensive hepatocyte degeneration, swelling, loose and light staining of cytoplasm, increased intercellular space, and hepatic congestion (Figure 6E; Supplementary Figure S2E). A large area of renal cells was replaced by proliferative connective tissue, with increased intercellular space accompanied by a large amount of lymphocyte infiltration (Figure 6F), a small amount of renal tubular epithelial cell degeneration, loose and light staining of cytoplasm (Supplementary Figure S2F). Both the immunosuppressed (Figures 6E,F) and the non-immunosuppressed groups (Supplementary Figures S2E,F) had strong lesions in liver and kidney. After PAS staining, spores and mycelia were abundant in both liver and kidney tissue (Figures 7E,F).

*Trichosporon inkin* caused a large amount of hepatocyte degeneration in mouse liver, with a small amount of lymphocyte infiltration, loose and light staining of cytoplasm, and a small amount of congestion (Figure 6G; Supplementary Figure S2G). A small number of renal tubular epithelial cells were degenerated in the field of renal section, with loose and lightly stained cytoplasm and a small amount of congestion (Figure 6H; Supplementary Figure S2H). Both the immunosuppressed (Figures 6G,H) and the non-immunosuppressed groups (Supplementary Figures S2G,H) had lesions but no obvious inflammatory changes. After PAS staining, spores were found in both liver and kidney tissue (Figures 7G,H).

*Trichosporon insectorum* caused a large amount of hepatocyte degeneration in mice, hepatocyte enlargement, loose cytoplasm and light staining, liver congestion (Figure 6I; Supplementary Figure S2I) and lymphocyte multifocal infiltration (Supplementary Figure S2I). A small number of degenerate renal tubular epithelial cells are observed, along with loose cytoplasm and light staining, congestion, and a small amount of lymphocyte infiltration (Figure 6J). In the immunosuppressed group (Figures 6I,J), there were strong lesions, while in the non-immunosuppressed group (Supplementary Figures S2I,J), the lesions mainly manifested in liver damage. A small number of spores and mycelia were observed in liver and kidney tissues after PAS staining (Figures 7I,J).

*Trichosporon japonicum* caused liver congestion in mice (Figure 6K; Supplementary Figure S2K), hepatocyte hyperplasia accompanied by inflammation, hepatocyte degeneration, loose and light staining of cytoplasm (Figure 6K). Many renal sacs were dilated and filled with eosinophilic substances, which were locally replaced by proliferative connective tissue with lymphocytic infiltrates (Figure 6L; Supplementary Figure S2L). The immunosuppressed group (Figures 6K,L) had obvious lesions, while the non-immunosuppressed group (Supplementary Figures S2K,L) mainly showed kidney damage. PAS staining revealed spores in liver and kidney tissue (Figures 7K,L).

## 4 Discussion

Microorganisms directly and indirectly impact reproductive success of animals (Stumpf et al., 2013; Pal, 2015; Ma et al., 2017). However, there have been no reports comparing the differences in the vaginal microbiome of giant pandas in estrus versus non-estrus. In this study, we analyzed the changes in mycobiota in the vagina of giant pandas in estrus and non-estrus conditions using ITS high-throughput sequencing, then isolated and identified potential pathogenic fungi, and experimentally explored their pathogenicity.

### 4.1 Analysis of high-throughput sequencing results during estrus and non-estrus in the giant panda

The vaginal mycobiota shares the same two abundant taxa at the phylum level between estrus and non-estrus species: Ascomycota and Basidiomycota. At the genus level, the top 10 fungal genera and their relative abundances were significantly different between estrus and non-estrus, and the fungal diversity during estrus showed a downward trend. The abundance of *Malassezia*, *Trichosporon*, *Apiotrichum*, and *Cutaneotrichosporon* increased during the estrus of giant pandas and became the main components of the vaginal mycobiota. The richness of *Trichosporon* and *Cutaneotrichosporon* was significantly higher in estrus than that observed in the non-estrus period, and the results of the LEfSe analysis were consistent. In addition, *Cladosporium* and *Vishniacozyma* were significantly less abundant compared to levels in the NEV group, whereas *Cladosporium* and *Vishniacozyma* are commonly found in the environment and in crops (Ghiaie Asl et al., 2017; Aleynova et al., 2022). Therefore, female giant pandas were hypothesized to be affected by hormones that induce the production of large amounts of vaginal secretions during estrus, which would reduce fungal colonization to a certain extent, thus decreasing the diversity of vaginal fungal species and increasing the abundance of dominant fungal genera during estrus. It was apparent that *Trichosporon*, *Apiotrichum*, and *Cutaneotrichosporon* could be the dominant fungal genera in the vagina of giant pandas during estrus.

*Trichosporon*, *Apiotrichum*, and *Cutaneotrichosporon* all belong to the *Trichosporonaceae* family, of which *Apiotrichum* and *Cutaneotrichosporon* were separated from *Trichosporon* by Liu et al. (2015) according to the principle of “one fungus = one name”. Zhang et al. conducted metagenomic sequencing of vaginal secretions of giant pandas during estrus, and the results showed that there were seven fungal genera among the top 35 genera, including *Trichosporon* and *Cutaneotrichosporon* (Zhang et al., 2020). Chen et al. analyzed the vaginal fungi of giant pandas of different living environments and ages using high-throughput sequencing and found *Trichosporon* to be one of the dominant genera (Chen et al., 2018). *Trichosporon* is the most abundant genus of all the microorganisms detected in the samples and is an important human and animal conditionally pathogenic yeast-like fungus that is widely distributed in nature. It can cause superficial and systemic infections (Chagas-Neto et al., 2008).

### 4.2 Isolation and identification of potentially pathogenic fungi

Seventeen potentially pathogenic strains were isolated from the 18 giant panda vaginal samples, including seven strains of *Apiotrichum brassicae* (formerly *Trichosporon brassicae*), one strain of *Cutaneotrichosporon moniliiforme* (formerly *Trichosporon moniliiforme*), and ten strains of *Trichosporon* spp. (two strains of *T. asteroides*, one strain of *T. inkin*, one strain of *T. insectorum*, and five strains of *T. japonicum*). The phylogenetic relationships among the *Trichosporon* spp. were very close. The similarity between *T. asahii* and *T. asteroides* in the ITS region was 99.3%, and only 2–3 bases in the ITS region differed (Sugita et al., 1999). *Trichosporon montevideense* and *T. domesticum* ITS domains are identical, and it is not possible to distinguish different *Trichosporon* spp. based on their domains or D1/D2 domains (Hashino et al., 2013). Molecular identification based on IGS1 sequence analysis allows the accurate identification of fungal species and has a greater ability to discriminate between related species than other regions, such as the ITS region (Sugita et al., 2002; Taverna et al., 2014). Therefore, in this study, ITS, D1/D2, and IGS1 sites were selected to jointly identify and construct a phylogenetic tree.

Seventeen strains of potentially pathogenic fungi were identified, including nine strains isolated from giant pandas at the Chengdu Research Base of Giant Panda Breeding and eight strains isolated from giant pandas at the Wolong China Giant Panda Garden. *Trichosporon* and *Apiotrichum* were the dominant genera at the two sites, accounting for 89 and 100% of the samples from both sites, respectively. The dominant species in the Chengdu Research Base of Giant Panda Breeding were *A. brassicae* and *T. japonicum*, each accounting for 33% of the isolated fungi. The dominant species in the Wolong China Giant Panda Garden was *A. brassicae*, which accounted for 50% of the isolated fungi, and was isolated from all four panda vaginal samples. This was followed by *T. japonicum*, accounting for 25% of the isolated fungi.

*C. moniliiforme*, *A. brassicae*, and *T. insectorum* are mostly isolated from the environment and food, and there are no reports on their pathogenicity (Prakash et al., 2018; Kuncharoen et al., 2020). *A. brassicae* was the dominant fungus in the Wolong China Giant Panda Garden and was isolated from multiple panda samples from the Chengdu Research Base of Giant Panda Breeding and the Wolong China Giant Panda Garden, which we speculate is attributable to environmental factors. *Trichosporon asteroides*, *T. inkin*, and *T. japonicum* have been reported to infect humans and animals frequently, causing fungemia, meningitis, urinary tract infections, and other diseases (Guo et al., 2019; Li et al., 2020; Santos et al., 2022). *Trichosporon asteroides* is one of the most important species to cause disseminated diseases in immunocompromised patients (Miceli et al., 2011). One study identified *Trichosporon* spp. in several vaginal samples of giant pandas. Moreover three species, including *T. japonicum*, *T. brassicae*, and *Trichosporon cutaneous*, were found in a giant panda that had been sterile for many years (Ma et al., 2017). However, whether *Trichosporon* spp. cause any harm to the giant panda vagina or are part of the normal fungal mycobiota of the reproductive tract has not been further studied.

### 4.3 Morphological development of potentially pathogenic fungi

By permitting characterization of the micromorphology of colonies, microculture of slides has important reference significance for the identification of *Trichosporon* (Colombo et al., 2011). Little research has been conducted on the morphology of *Trichosporon*, *Apiotrichum*, and *Cutaneotrichosporon*. In 2005, Li et al. conducted molecular and morphological analyses of six strains of *Trichosporon* spp., among which the morphology of *T. inkin* was consistent with that of *T. inkin* isolated in our study (Li et al., 2005). Macroscopic colonies of different *Trichosporon* spp. were similar; however, the microscopic structure of the mycelia and spore morphology were significantly different. Under a microscope, septate hyphae, arthrospores, pseudohyphae, and blastospores were observed. Some *Trichosporon* spp. showed a characteristic structure; for example, *T. inkin* showed antler hyphae under a microscope, and *T. japonicum* hyphal fragmentation produced a large number of columnar arthrospores. *Apiotrichum* and *Cutaneotrichosporon* took longer to grow than *Trichosporon*. In this study, *Trichosporon* showed a large number of hyphae or arthrospores on day 2 of culture, whereas *A. brassicae* showed only conidial morphology and no hyphae production within 4 days of culture. In *C. moniliiforme* on day 4 of culture, the conidia showed a spindle shape with only a few short hyphae.

### 4.4 Pathogenicity of potentially pathogenic fungi

The pathogenicity of *Trichosporon*, *Apiotrichum*, and *Cutaneotrichosporon* was studied in mice. *Trichosporon* is considered a strain that colonizes or even infects the skin or mucous membranes and infects the body by barrier disruption caused by themselves or trauma (such as catheter implantation) (Mariné et al., 2015b). *Trichosporon inkin*, *T. ovoides* and *T. loubieri* are considered the most important species involved in superficial trichosporosis, whereas *T. asahii*, *T. asteroides*, and *T. japonicum* have been associated with aggressive infections in immunocompromised patients; deep infections with *T. inkin* have also been found (Silvestre Junior et al., 2010; Thion et al., 2017). Francisco et al. collected 24 *Trichosporon* clinical isolates from urine and blood samples collected from 358 medical centers, with *T. asahii* being the most common species (76.3%), followed by *T. inkin* (9.7%)(Francisco et al., 2019).

In this study, all fungi caused varying degrees of damage to the liver and kidneys of healthy mice, and the lesions were more pronounced in the immunosuppressed group than in the non-immunosuppressed group. Most PAS-stained spores were also observed in the liver and kidney sections, indicating that these strains could colonize the liver and kidneys and cause damage there. Among these, *C. meliforme* was the least pathogenic, while *T. asteroides* was the most pathogenic. *Trichosporon asteroides* non-immunosuppressive group survived, and the mortality rate in the immunosuppressive group reached 50%. The predominant pathological symptoms are that the liver and kidney cells are replaced by proliferative connective tissue, and there is a large amount of liver cell degeneration and swelling, and a small amount of renal tubular epithelial cell degeneration. Mice infected with other fungi showed decreased activity, but no change in mortality. Marine et al. found that different strains of *T. asteroides* had different degrees of pathogenicity in mice, with *T. asteroides* being less pathogenic than *T. asahii*, but more pathogenic than *T. inkin*; the conidia and hyphae of these three fungi could diffusely infiltrate the kidneys of mice (Mariné et al., 2015a). Jiang et al. studied the pathogenicity of *Trichosporon* isolated from the body surface of a giant panda in mice and found that *T. asteroides* (JYZ1255) was highly pathogenic, with 85% mortality, liver tissue hyperemia, and necrosis in immunosuppressed mice (Ma et al., 2019). In this experiment, compared to JYZ1255, the mortality rate of *T. asteroides* was lower when inoculated with the same concentration of bacterial solution, and its pathogenicity may be lower than that of JYZ1255.

*Trichosporon japonicum* is a very rare cause of invasive trichosporosis and can be isolated from various sources such as biopsy, skin, sputum, vaginal mucosa, bile, urine, pleural effusion, and fingernails (Kalkanci et al., 2010; do Espírito Santo et al., 2020). In this study, after inoculation with *T. japonicum*, the liver and kidney tissues of mice in the immunosuppressed group were damaged, but those in the non-immunosuppressed group were mainly damaged in the kidney. It was speculated that the main site of damage of *T. japonicum* was the kidneys.

Female giant pandas are hormonally affected during estrus, producing large amounts of vaginal secretions. These secretions serve as a protective barrier, preventing the colonization of external and intrinsic fungi in the vagina, resulting in a reduction in the diversity and an increase in the specificity of the vaginal mycobiota (Martin-Wintle et al., 2019). Compared with that in the NEV group, the diversity of fungal communities was reduced in the EV group. Thus, it was hypothesized that vaginal secretions during the estrous period of giant pandas reduce the colonization of some fungi and increase the proportion of dominant species. Moreover, estrogen promotes the keratinization of vaginal epithelial cells, thereby providing a favorable environment for fungal colonization (Miao et al., 2023). After binding to host cells, fungi can cause epithelial cell damage, change the vaginal pH, and modulate host immune responses, thereby resulting in the manifestation of infection symptoms, such as itching, redness, swelling and mucus discharge (Parolin et al., 2015; Sheppard, 2020; d’Enfert et al., 2021). Moreover, the altered physiologic state of the vaginal environment has important implications for both host health and reproduction.

The vaginal mycobiota of giant pandas is greatly affected by the environment, animal age, and estrus; however, *Trichosporon* has been identified in many studies, suggesting that it might be a resident of the vaginal mycobiota of giant pandas and is the dominant mycobiota during estrus. It is well known that *Trichosporon* is a conditionally pathogenic bacterium that increases the chance of trichosporosis disease when immunity is suppressed; thus, improving the immunity of giant pandas during the estrous period is an important way to prevent this disease. This shows that the temporary changes in the vaginal mycobiota that occur during estrus in female giant pandas are also potentially harmful, and it is of great interest to explore the pathogenicity of potentially pathogenic bacteria during estrus. However, surveys based on giant panda vaginal samples are limited by the timing of estrus and the number of giant pandas, and therefore, large numbers of samples and appropriate sampling times are expected to yield more comprehensive data on vaginal fungal communities.

## 5 Conclusion

The vaginal secretions of giant pandas during estrus play a dominant role in fungal colonization, and the diversity of the vaginal mycobiota of giant pandas in estrus decreases and specificity increases. *Malassezia*, *Trichosporon*, *Apiotrichum*, and *Cutaneotrichosporon* were the dominant genera in the vaginal mycobiota of giant pandas during estrus. Moreover, the richness of *Trichosporon* and *Cutaneotrichosporon* in the vaginal mycobiota of giant pandas was significantly higher during estrus than during non-estrus periods. *Apiotrichum* and *Cutaneotrichosporon* were considered the most important genera, primarily originating from the environment and caused by marking behavior during the estrous period, in giant pandas. *Trichosporon* is considered a resident mycobiota of the vagina and is an important pathogen that infects the organism when immunity is suppressed with *T. asteroides* being the most pathogenic, resulting in extensive connective tissue replacement and inflammatory cell infiltration in both liver and kidney tissues. The results of this study improve our understanding of the diversity of the vaginal fungi present in giant pandas and will significantly contribute to improving the reproductive health of giant pandas in the future.

## Data availability statement

The datasets presented in this study can be found in online repositories. The names of the repository/repositories and accession number(s) can be found in the article/Supplementary material.

## Ethics statement

The animal study was approved by the Institutional Animal Care and Use Committee of Sichuan Agricultural University (permit number DYY-S20183033). The study was conducted in accordance with the local legislation and institutional requirements.

## Author contributions

XM: Conceptualization, Funding acquisition, Project administration, Supervision, Writing – original draft, Writing – review & editing. ZL: Data curation, Formal analysis, Investigation, Software, Writing – original draft. CY: Conceptualization, Data curation, Funding acquisition, Investigation, Project administration, Supervision, Writing – review & editing. SW: Investigation, Writing – original draft. XL: Data curation, Formal analysis, Methodology, Writing – original draft. CW: Investigation, Methodology, Visualization, Writing – review & editing. ShL: Formal analysis, Investigation, Methodology, Resources, Writing – review & editing. YW: Formal analysis, Investigation, Methodology, Validation, Writing – review & editing. SoL: Resources, Software, Supervision, Validation, Writing – review & editing. YG: Data curation, Investigation, Methodology, Software, Validation, Writing – review & editing.
