# Supplementary material for: High-throughput sequencing and characterization of potentially pathogenic fungi from the vaginal mycobiome of giant panda (Ailuropoda melanoleuca) in estrus and non-estrus
Source: Front Microbiol. 2024 Jan 25;15:1265829. doi: 10.3389/fmicb.2024.1265829 (PMC10850575; doi:10.3389/fmicb.2024.1265829)
Supplement: Supplementary file 1 [file Table_1.DOCX]

Supplementary Material

Supplementary Table 1 Sample group information

| Number | Group | Location |
| --- | --- | --- |
| EV1 | EV | Chengdu Research Base of Giant Panda Breeding |
| EV2 | EV | Chengdu Research Base of Giant Panda Breeding |
| EV3 | EV | Chengdu Research Base of Giant Panda Breeding |
| EV4 | EV | Chengdu Research Base of Giant Panda Breeding |
| EV5 | EV | Chengdu Research Base of Giant Panda Breeding |
| EV6 | EV | Chengdu Research Base of Giant Panda Breeding |
| EV7 | EV | Chengdu Research Base of Giant Panda Breeding |
| EV8 | EV | Chengdu Research Base of Giant Panda Breeding |
| EV9 | EV | Chengdu Research Base of Giant Panda Breeding |
| NEV1 | NEV | Chengdu Research Base of Giant Panda Breeding |
| NEV2 | NEV | Chengdu Research Base of Giant Panda Breeding |
| NEV3 | NEV | Chengdu Research Base of Giant Panda Breeding |
| NEV4 | NEV | Chengdu Research Base of Giant Panda Breeding |
| NEV5 | NEV | Chengdu Research Base of Giant Panda Breeding |
| NEV6 | NEV | Chengdu Research Base of Giant Panda Breeding |
| WL1 | / | Wolong Giant Panda Breeding Center |
| WL2 | / | Wolong Giant Panda Breeding Center |
| WL3 | / | Wolong Giant Panda Breeding Center |
| WL4 | / | Wolong Giant Panda Breeding Center |
| WL5 | / | Wolong Giant Panda Breeding Center |
| WL6 | / | Wolong Giant Panda Breeding Center |
| WL7 | / | Wolong Giant Panda Breeding Center |
| WL8 | / | Wolong Giant Panda Breeding Center |
| WL9 | / | Wolong Giant Panda Breeding Center |

Supplementary Table 2 Nucleotide sequence accession numbers

| Strain | Molecular Identification | ITS GenBank  Accession Number | D1/D2 GenBank Accession Number | IGS1 GenBank  Accession Number |
| --- | --- | --- | --- | --- |
| WSW66 | *T.* *asteroides* | MW426439 | MW454143 | MW454083 |
| WSW88 | *T.* *asteroides* | MW433660 | MW454338 | MW543944 |
| WSW77 | *A.* *brassicae* | MW433664 | MW454341 | MW433665 |
| WSW013 | *A. brassicae* | MW433671 | MW454373 | MW433672 |
| WSW019 | *A. brassicae* | MW440469 | MW463372 | MW440470 |
| WSW021 | *A. brassicae* | MW440473 | MW463388 | MW463389 |
| WSW006 | *A. brassicae* | MW449593 | MW463398 | MW449594 |
| WSWW26 | *A.* *brassicae* | MW450473 | MW466636 | MW450474 |
| WSWS06 | *A. brassicae* | MW449583 | MW466576 | MW449584 |
| WSW020 | *T. japonicum* | MW440471 | MW463374 | MW440472 |
| WSW023 | *T. japonicum* | MW440475 | MW463390 | MW440476 |
| WSWW2 | *T. japonicum* | MW449606 | MW466599 | MW449607 |
| WSWW19 | *T. japonicum* | MW449612 | MW466632 | MW449613 |
| WSW018 | *T. japonicum* | MW440466 | MW463368 | MW440467 |
| WSW014 | *T. inkin* | MW433673 | MW454381 | MW433674 |
| WSW001 | *C.* *moniliiforme* | MW449581 | MW463396 | MW449582 |
| WSW010 | *T.* *insectorum* | MW433666 | MW454343 | MW433667 |

Supplementary Figure 1. Anatomical changes in KM mice after 7 days.

**(A)** The abdominal cavity contains many ascites. **(B)** White nodules appear in the liver.

Supplementary Figure 2. Representative HE stained sections of liver **(A、C、E、G、I、K)** and kidney **(B、D、F、H、J、L)** tissues of non-immunosuppressed mice on the 7th day after infection with *C. moniliiforme*, *A. brassicae*, *T. asteroid, T. inkin*, *T. insectorum* and *T. japonicum*, respectly. The spores and mycelia are indicated with arrows. Scale bars, 100 μm.
