# Supplementary figures and images for: High-throughput sequencing and characterization of potentially pathogenic fungi from the vaginal mycobiome of giant panda (Ailuropoda melanoleuca) in estrus and non-estrus
Source: Front Microbiol. 2024 Jan 25;15:1265829. doi: 10.3389/fmicb.2024.1265829 (PMC10850575; doi:10.3389/fmicb.2024.1265829)

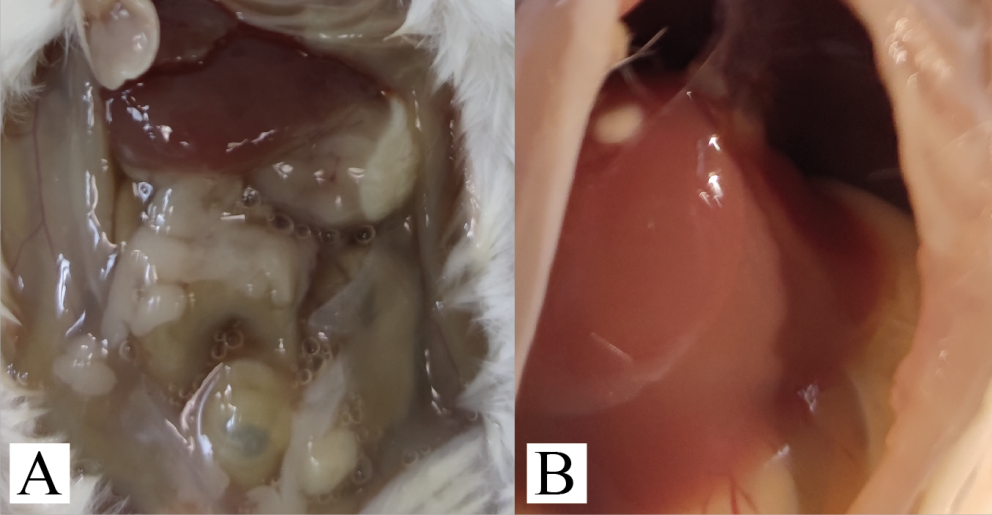

Supplement: Supplementary file 2 [file Image_1.PNG]

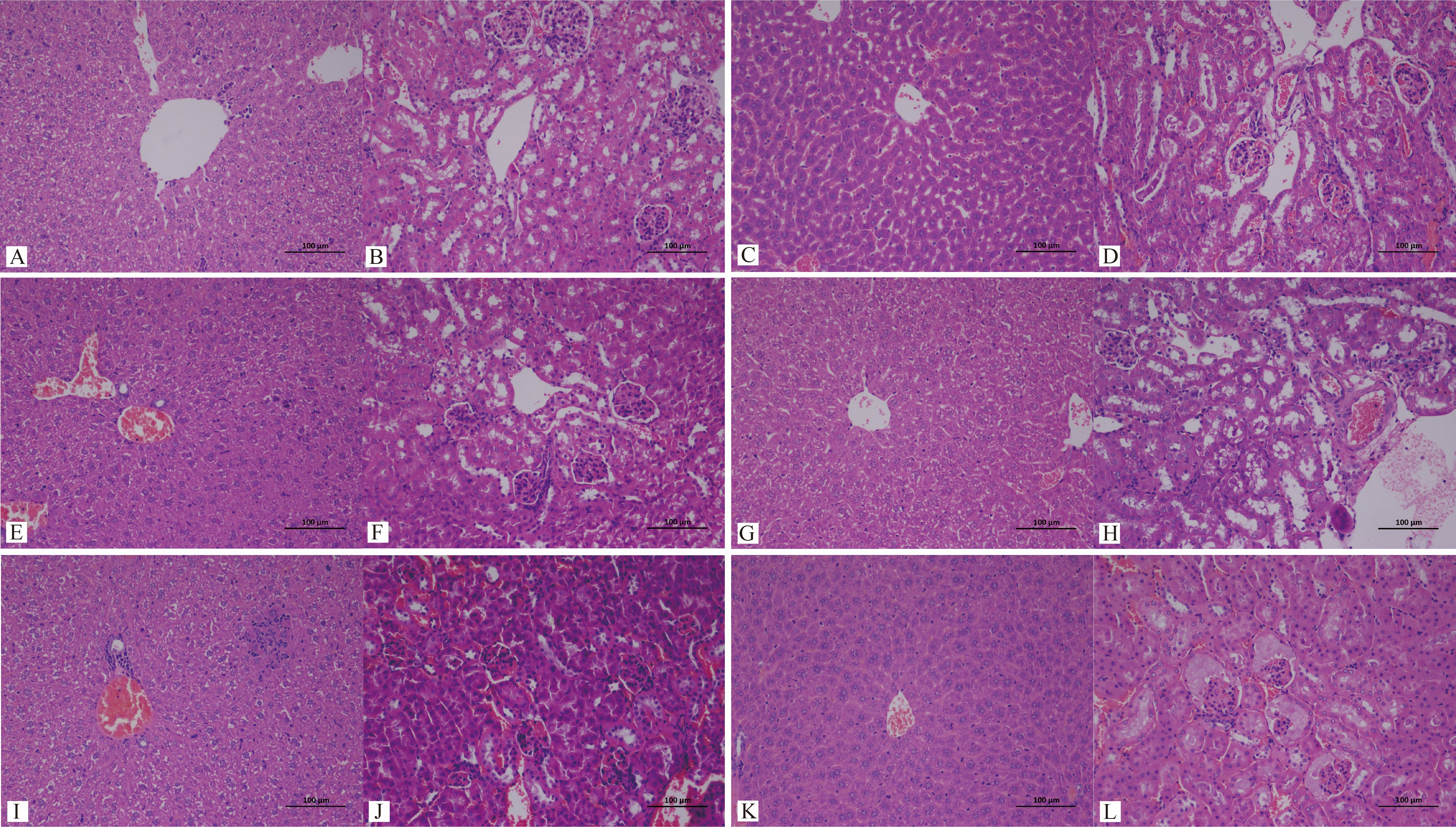

Supplement: Supplementary file 3 [file Image_2.JPEG]
